# Cross-Sectional Associations of Body Adiposity, Sedentary Behavior, and Physical Activity with Hemoglobin and White Blood Cell Count

**DOI:** 10.3390/ijerph192114347

**Published:** 2022-11-02

**Authors:** Tiia Koivula, Salla Lempiäinen, Saara Laine, Tanja Sjöros, Henri Vähä-Ypyä, Taru Garthwaite, Eliisa Löyttyniemi, Harri Sievänen, Tommi Vasankari, Juhani Knuuti, Ilkka H. A. Heinonen

**Affiliations:** 1 Turku PET Centre, University of Turku and Turku University Hospital, 20520 Turku, Finland; 2 Oncology Clinic, Turku University Hospital, 20520 Turku, Finland; 3 The UKK-Institute, 33500 Tampere, Finland; 4 Department of Biostatistics, University of Turku, 20520 Turku, Finland; 5 Rydberg Laboratory of Applied Sciences, University of Halmstad, 30118 Halmstad, Sweden

**Keywords:** accelerometer, inflammatory markers, abnormal blood count, overweight, physical activity, sedentary behavior

## Abstract

Background: This study examined whether hemoglobin (Hb) and white blood cell count (WBC) associate with body adiposity and other cardiometabolic risk factors, as well as accelerometer-measured sedentary behavior (SB) and physical activity (PA), when adjusted for body mass index (BMI). Methods: The cross-sectional analysis included 144 participants (42 men) with a mean age of 57.0 years and a mean BMI of 31.7 kg/m^2^. SB and standing time, breaks in sedentary time and PA were measured during four consecutive weeks with hip-worn accelerometers. A fasting blood sample was collected from each participant during the 4-week measurement period and analyzed using Sysmex XN and Cobas 8000 c702 analyzers. Associations of WBC, Hb and other red blood cell markers with cardiometabolic risk factors and physical activity were examined by Pearson’s partial correlation coefficient test and with linear mixed regression models. Results: In sex- and age-adjusted correlation analyses both BMI and waist circumference correlated positively with Hb, WBC, red blood cell count (RBC), and hematocrit. Hb was also positively correlated with systolic blood pressure, insulin resistance scores, liver enzymes, LDL, and triglyceride levels. Sedentary time correlated positively with WBC, whereas standing time correlated negatively with WBC. Lying time correlated positively with WBC, RBC, hematocrit, and Hb. Regarding SB and PA measures, only the association between lying time and RBC remained significant after adjustment for the BMI. Conclusion: We conclude that body adiposity, rather than components of SB or PA, associates with Hb levels and WBC, which cluster with general metabolic derangement.

## 1. Introduction

Hemoglobin (Hb) is an iron-containing oxygen transport metalloprotein in the red blood cells of almost all vertebrates. Hb carries oxygen in blood from the lungs to the tissues, where oxygen is released to permit aerobic respiration for energy provision to metabolic processes [1]. Obesity is defined as excess adipocyte mass in the body, but also, dysfunctional changes in the obese adipose tissue are evident as compared to lean adipose tissue. For example, macrophage infiltration and local production of proinflammatory cytokines create a low-grade systemic inflammatory milieu in the adipose tissue [2]. Metabolic syndrome, currently prevalent in 20–25% of the world’s adult population, refers to the co-occurrence of several known cardiovascular risk factors including obesity, insulin resistance, atherogenic dyslipidemia, and hypertension [3]. Increasing evidence indicates that also elevated serum ferritin levels independently predict type 2 diabetes mellitus [4]. Elevated ferritin levels have also been associated with hypertension [5], dyslipidemia [6], elevated fasting insulin and blood glucose levels [7], central adiposity [8], and metabolic syndrome [9]. Higher Hb and hematocrit (HTC) levels have also been observed to associate with increased insulin resistance, hypertension, hypercholesterolemia, and hypertriglyceridemia in healthy populations [10,11,12,13]. It has been suggested that hyperviscosity of plasma or changes in plasma volume are mediators in these associations [12]. However, this might be a too simplistic view, since the commonly observed high levels of, e.g., insulin or triglycerides in obese persons may not be simply due to a lower plasma volume in these individuals. Recently, it has been noticed that the tissue oxygenation status may indeed be associated with metabolic health [14,15]. Still, not enough is known about the connections between obesity and Hb and other red blood cell markers, and their clustering with other traditional cardiometabolic risk factors, especially among individuals with overweight and obesity.

Increased body adiposity is often associated with a higher amount of daily sitting and little physical activity (PA), even after adjustments for numerous confounding factors such as diet and genetic predisposition to obesity [16]. Many studies have also shown unfavorable associations between the total amount of accelerometer-measured sedentary time and metabolic health outcomes, including cholesterol and triacylglycerol levels, markers of insulin resistance and metabolic syndrome [17,18,19]. A sedentary lifestyle has also been shown to be related to impaired kidney function in many cross-sectional studies [20]. The pattern of sedentary time, i.e., the frequency of interruptions to sedentary time (breaks), could also be relevant to health outcomes [21,22,23], but overall, the role of sitting and physical activity habits have been fairly sparsely investigated in terms of Hb, erythrocyte characteristics, and leukocytes. A few previous studies have demonstrated a connection between sedentary behavior (SB) and inflammatory markers [24,25]. Breaks in the sedentary time have also been associated with improved the influence on the procoagulant effects of uninterrupted sitting in sedentary overweight and obese adults [26]. However, to the best of our knowledge, the role of sitting and physical activity habits in determining red blood cell characteristics, as well as levels of Hb, HTC, leukocytes, and thrombocytes (TC), is incompletely characterized, especially independently of body adiposity.

Along these lines, the primary aim of the present study was to verify whether body adiposity (BMI and WC, in this context) correlates positively with Hb and other red blood cell markers and whether these blood markers are clustered with classical cardiometabolic risk factors independently of body adiposity. Secondly, we aimed to investigate whether the total amount of daily sitting, lying, breaks in sitting time, standing, or light or moderate-to-vigorous physical activity are associated with Hb, red blood cell characteristics, and leukocytes, especially after adjusting for body adiposity. 

## 2. Materials and Methods

This study was a single-arm observational study conducted at the Turku PET Centre, Turku, Finland, between April 2017 and May 2019. This study is part of a larger trial (Clinicaltrials.gov ID NCT03101228). Informed consent was obtained from the participants before entering the study, and good clinical practice and the Declaration of Helsinki were followed. The study was approved by the Ethics Committee of the Hospital District of Southwestern Finland (16/1810/2017).

### 2.1. Participants 

As previously reported [27,28], the participants in this study were recruited from the local community by newspaper advertisements and bulletin leaflets. Inclusion criteria were the following: age 40–65 years, BMI 25–40 kg/m^2^ and self-reported insufficient physical activity to meet the current recommendations at the time and sitting for major proportion of the day. The exclusion criteria were the following: history of a cardiac event, insulin- or medically treated diabetes, abundant use of alcohol according to national guidelines, use of narcotics, smoking of tobacco or consuming of snuff tobacco, inability to understand written Finnish and any chronic disease or condition that could create a hazard to the participant’s safety or endanger the study procedures. All the eligible participants that volunteered during the recruitment period were included in this study.

### 2.2. Study Design

The eligible volunteers were interviewed and provided with an accelerometer, which they were instructed to wear on the right hip for four consecutive weeks, starting from the following morning. The participants were instructed to wear the accelerometer during waking hours, except for activities where the device could be exposed to water. Moreover, they were advised to maintain their usual physical activity habits during the measurement. During the 4-week measurement period, the participants were asked to visit the laboratory once for fasting venous blood samples at their most convenient time [27,28].

### 2.3. Anthropometrics

All the anthropometric outcomes were measured during the recruitment interview under standard conditions and by the same researcher to avoid measurement bias. Participants chose the hour of the day according to their convenience. As previously reported [27,28], blood pressure and resting heart rate were measured with a digital blood pressure monitor (Apteq AE701f, Rossmax International Ltd., Taipei, Taiwan) in a seated position after at least 10 min of sitting. The mean of 2 to 3 measurements was used as the outcome measure. Body weight was measured by a scale (Seca 797, Vogel & Halke, Hamburg, Germany) in light clothing, and body height was measured barefoot with a wall-mounted stadiometer (KaWe person check, Kirchner & Wilhelm GmbH + Co., Asperg, Germany). WC was measured with a flexible measuring tape midline between the iliac crest and the lowest rib, and the measurement was repeated at least twice. 

### 2.4. Sedentary Behavior and Physical Activity Measurements

SB and PA were measured for four consecutive weeks with a hip-worn triaxial accelerometer (UKK AM30, UKK-Institute, Tampere, Finland), as reported and described in detail in our previous publications [27,28,29,30,31]. 

In summary, the collected accelerometer data was analyzed in 6-s epochs using a validated mean amplitude deviation (MAD) method, and the epoch-wise MAD values were converted to metabolic equivalents (METs; 1 MET = 3.5 mL/kg/min of oxygen consumption). Sitting, lying and standing were defined as <1.5 METs. In order to differentiate between the three different behaviors, body posture was assessed by the validated angle for posture estimation (APE) method, which identifies postures with 90% accuracy in free living conditions. Breaks in sedentary time were determined as SB periods with a one-minute exponential moving average <1.5 METs, ending in vertical acceleration and subsequent standing posture or movement. Light PA (LPA) was defined as 1.5–2.9 METs and moderate-to-vigorous PA (MVPA) as ≥3 METs. The amount of vigorous PA (≥6.0 METs) among participants was negligible; thus, moderate and vigorous PA were combined as MVPA. The total amount of SB was calculated by adding sitting and lying together, and the total PA was calculated by adding LPA to MVPA. In addition to the absolute time spent in each behavior (h/day), the daily proportions of different behaviors (SB, standing, LPA, MVPA) were calculated and presented as percentage of the accelerometer wear time. Additionally, the epoch-wise MET values were further smoothed with a 1-min exponential moving average, and the mean of the daily peak MET values and the mean of the daily mean MET values of the smoothed epoch-wise MET values were calculated. Wear times of 10–19 h/day and at least four days of measurements were considered valid. The accelerometer data analysis methods are described in more detail in our previous publications [27,28,29,30,31].

### 2.5. Blood Biomarkers 

As previously reported [27,28], venous blood samples were drawn after at least 10 h of fasting and analyzed at the Turku University Hospital Laboratory. Fasting blood samples were analyzed using standard assays. White blood cell count was analyzed using the flow cytometry method (Sysmex XN analyzer, Sysmex, Kobe, Japan). Red blood cell count and thrombocytes were determined by the hydrodynamically focused DC detection method (Sysmex XN analyzer, Sysmex, Kobe, Japan). Hematocrit was calculated via the RBC pulse height detection method (Sysmex XN analyzer, Sysmex, Kobe, Japan). Hb was determined by the SLS method (Sysmex XN analyzer, Sysmex, Kobe, Japan). Mean cell volume (MCV) was calculated by dividing HTC by RBC. Similarly, mean corpuscular hemoglobin (MCH) was calculated by dividing Hb by RBC. Creatinine (Cr) was determined by the enzymatic method (Cobas 8000 c702 Analyzer, Roche Diagnostics GmbH, Mannheim, Germany). Alanine aminotransferase (ALT) and aspartate aminotransferase (AST) were determined by the photometric (IFCC) method (Cobas 8000 c702 Analyzer, Roche Diagnostics GmbH, Mannheim, Germany). Gamma-glutamyl transferase (GGT) was determined with the enzymatic colorimetric assay (Cobas 8000 c702 Analyzer, Roche Diagnostics GmbH, Mannheim, Germany). Plasma insulin was analyzed using the electrochemiluminescence immunoassay (Cobas 8000 e801, Roche Diagnostics GmbH, Mannheim, Germany). Plasma glucose was determined by the enzymatic reference method with hexokinase GLUC3 and plasma triglycerides and total, LDL and HDL cholesterol by enzymatic colorimetric tests (Cobas 8000 c702, Roche Diagnostics GmbH, Mannheim, Germany). The HOMA-IR index was calculated using the formula: fasting glucose × fasting insulin/22.5. HbA_1c_ was determined by the turbidimetric inhibition immunoassay (Cobas 6000 c501, Roche Diagnostics GmbH, Mannheim, Germany). 

### 2.6. Statistical Methods 

The associations were examined with Pearson’s partial correlation coefficient test and linear mixed regression models. In the regression models, the associations between blood parameters (dependent variables) and anthropometrics, cardiometabolic risk factors, SB and PA (independent variables) were adjusted for one categorical (sex) and one continuous variable (age) (model 1). For further analyses, one continuous variable (BMI) (model 2) was added to the model to control for the confounding overweightness. Sex differences were analyzed with the *t*-test or Fisher’s exact test. The normal distributions of the residuals were examined visually by the normal quantile plot and Shapiro–Wilks test. Logarithmic (log10) transformations were performed when necessary to achieve normal distribution of the data. Missing data were handled by pairwise deletion. If not otherwise stated, data were expressed as the mean and standard deviation (SD). The level of statistical significance was set at 5% (two-tailed). All analyses were carried out with JMP pro 13.1 for Windows (SAS Institute Inc., Cary, NC, USA) and IBM SPSS Statistics for Windows, Version 27.0 (IBM Corp, Armonk, NY, USA).

## 3. Results 

In total, 263 participants volunteered, of whom 102 women and 42 men were eligible and completed the accelerometer measurements. Out of 144 participants, two did not have fasting blood samples and another two resting heart rate values. According to the national classification [32], 55 of the participants were overweight (BMI ≥ 25 kg/m^2^), and 87 were obese (BMI ≥ 30 kg/m^2^). All participants had WC over cut-off values (94 cm for men and 80 cm for women [33]). The mean accelerometer wear time was 14.37 (SD 1.04) h/day, and the mean duration of the measurement was 25 (SD 4) days. The participants spent 67.0 (SD 8.3)% of the accelerometer wear time in sedentary activities and took, on average, 5265 (SD 2113) steps per day. The RBC, HTC and Hb levels differed statistically significantly between men and women. Sex differences were also observed in the WC, insulin, HOMA-IR, ALT, GGT, cholesterol, and HDL levels, as well as in SB time and SB proportion, lying time, breaks in sedentary time, and standing time and proportion. There were no significant differences between sexes in any PA variables (Table 1). 

### 3.1. Blood Parameters, Anthropometrics and Cardiometabolic Risk Factors 

In sex- and age-adjusted correlation analyses, we found that both BMI and WC associated positively with Hb, WBC, RBC and HTC and negatively with Cr (Table 2). Hb was also positively associated with lower systolic blood pressure (SBP), HOMA-IR, ALT, GGT, LDL, and triglyceride levels, when adjusted for age and sex (Table 2).

Additionally, HTC associated positively with both the SBP and diastolic blood pressure (DBP), HOMA-IR scores, ALT, GGT, and LDL levels and negatively with the HDL levels (Table 2). Positive associations were also found between RBC and SBP and DBP and HOMA-IR and ALT levels (Table 2). The WBC levels showed positive associations with fasting glucose, insulin, HOMA-IR, GGT, and triglyceride levels and a negative association with HDL (Table 2). 

Cr associated negatively with the SBP and GGT levels (Table 2), while a positive association was found between the MCH and GGT levels, as well as between TC and blood pressure medication (Table 2). All the above-mentioned results were adjusted for sex and age.

For further analysis, we ran multivariable regression analyses, including BMI as an additional covariate, to see whether the blood Hb and RBC and WBC parameters are clustered with the classical cardiometabolic risk factors independently of adiposity. An adjustment for BMI attenuated most of the associations. However, the associations of Hb and HTC with LDL remained significant. Additionally, the associations of RBC and both SBP and DBP and the associations between WBC and HOMA-IR and triglycerides remained significant. Moreover, the associations between Cr and SBP, as well as between TC and blood pressure medication remained significant. Additionally, the associations of MCV and MCH with blood pressure medication turned significant when further adjusted for BMI (Table 3).

### 3.2. Blood Parameters and SB and PA 

The results from the sex- and age-adjusted correlation analyses showed significant associations between the greater sedentary proportion of the accelerometer wear time (%/day) and higher WBC. Additionally, we found a negative association between the standing time and WBC. The lying time was associated positively with WBC, RBC, HTC, and Hb and negatively with MCV, with breaks in the sedentary time (n/day) associated positively with Cr, while the proportion of LPA associated positively with MCV (Table 4).

A further analysis showed that adding BMI to the model had a major impact on the results. The associations between lying time and RBC, as well as the association between LPA proportion and MCV, remained significant when adjusted for sex, age and BMI, but all other associations turned nonsignificant. However, when the BMI was added to the model, the association between breaks in sedentary time and Hb turned significant. Moreover, the associations between LPA and MCV, as well as the association between the LPA proportion and MCH, turned significant when additionally adjusted for the BMI (Table 5).

## 4. Discussion

The aim of this study was to verify whether body adiposity (BMI and WC) correlates positively with Hb and other red blood cell markers and whether these markers are clustered with classical cardiometabolic risk factors independently of adiposity. We also aimed to investigate whether the total amount of daily sitting, lying, standing, LPA or MVPA or breaks in sedentary time were associated with Hb, red blood cell characteristics and leukocytes, especially after adjusting for the body adiposity. 

In the present study, we found that both BMI and WC associated positively with Hb, WBC, RBC, and HTC and negatively with Cr when adjusted for age and sex. Participants with lower Hb levels had significantly lower SBP, HOMA-IR scores, ALT, GGT, LDL, and triglyceride levels. HTC and RBC associated positively with both SBP and DBP, HOMA-IR scores and ALT. WBC levels showed a positive association with fasting glucose, fasting insulin, HOMA-IR scores, GGT, and triglyceride levels and a negative association with HDL levels. Further adjustment for BMI attenuated most of the associations. We also observed an association between SB and increased WBC, RBC, Hb, and HTC. Greater proportion of LPA was associated with an increased average volume of red blood cells. However, most of these associations with SB and PA components turned nonsignificant when adjusted for BMI. Altogether, these results suggest that body adiposity is an important mediator of clustering of blood count variables with common cardiometabolic risk factors, as well as their relations to SB and PA. 

Driven by the previous finding that tissue oxygenation status may associate with metabolic health [14,15], a Finnish group investigated the possibility of using Hb levels as a surrogate marker of body oxygenation and studied its association to metabolic health [34]. They used genetically altered mice and found a positive association between Hb levels, body weight, glucose tolerance, and HOMA-IR scores. The association of Hb levels in humans was then examined by the same group by running cross-sectional and longitudinal analyses in two populations: Northern Finland Birth Cohort 1966 and Cardiovascular Risk in Young Finns Study (YFS; total *n* = 7175). A strong positive correlation between the Hb levels and BMI was found, as well as positive associations between the Hb levels and fasting glucose and insulin levels, as well as insulin resistance indexes (i.e., HOMA-IR), similar to the findings in mice. Their study also showed positive association between Hb and SBP and DBP, serum total cholesterol, LDL cholesterol, triglycerides, and C-reactive protein (CRP) and a negative association between Hb and HDL cholesterol. The associations were attenuated when adjusted for BMI but, apart from CRP, remained significant [34]. The group also studied the activation of hypoxia-induced genes in a subgroup of individuals in the YFS study and found a difference in the transcriptional activation of these genes between the lowest and the highest quartiles of Hb (<132 g/L and >152 g/L, respectively) [34]. As Auvinen et al. showed, Hb correlates positively with the BMI, and the results in our observational study are well in accordance with these results [34]. The study by Auvinen et al. also indicated that Hb associates positively with the fasting glucose and insulin levels and insulin resistance index (i.e., HOMA-IR), similarly to our finding of the association between low Hb and more beneficial HOMA-IR scores. Similar results have also been found by Hämäläinen et al. [35]. They showed that individuals with metabolic syndrome had elevated Hb, ferritin, erythropoietin, and haptoglobin concentrations [35]. Higher Hb levels were related to all the components of metabolic syndrome, including abdominal obesity, increased blood pressure, glucose intolerance, and dyslipidemia. Our results are well in line with these results, and overall, the evidence suggests that abdominal obesity is an important determinant of higher Hb levels in adults without diagnosed cardiometabolic diseases. Chronic inflammation due to obesity also alters the size variations of circulating red blood cells [36]. In the present study, we did not analyze the red blood cell distribution width (RDW), but previously other studies have shown that obesity and chronic inflammation are also linked with increased RDW [36], and it is thus considered a biomarker for the prognosis of many diseases.

The mechanisms that could explain the associations between cardiovascular disease risks and higher Hb levels are not yet well understood. Whether Hb could serve as a surrogate marker for metabolic syndrome is also to be proven, but our study suggests that there is a correlation. It is namely known that living at a higher altitude, where the arterial oxygen saturation is not complete as it normally is at the sea level, associates favorably with obesity risks, diabetes and numerous traditional cardiovascular risk factors [37,38,39]. Lower Hb levels, similar to reduced arterial oxygenation at high altitudes, may trigger a minor hypoxic response even at the sea level, which mediates the beneficial effects on metabolic health. In this regard, it is known that when tissues encounter reduced oxygen levels, such as at altitude, hypoxia-inducible factor (HIF) becomes stabilized. This upregulates genes that regulate the energy metabolism. During the last few years, studies have investigated specific prolyl 4-hydroxylases (P4Hs) that regulate the stability of HIF, a potent governor of metabolism. Recent studies showed that the inhibition of HIF-P4Hs protects mice from obesity, metabolic syndrome and associated diseases [40,41]. Additionally, HIF-P4H-2-deficient mice had less adipose tissue, smaller adipocytes and less adipose tissue inflammation than the control mice, regardless of diet [15]. They also had improved glucose tolerance and insulin sensitivity and decreased serum cholesterol and de novo lipid synthesis compared to the controls, and the mice were protected against hepatic steatosis. Moreover, mice with this deficient gene had better glucose tolerance and HOMA-IR than control mice, and also, the mass of white adipose tissue was smaller with a reduction in adipocyte size [14]. Furthermore, macrophage infiltration into white adipose tissue was eased. 

Further, it is known that insulin has synergistic effects on stimulating erythrocyte production together with erythropoietin [42]. Thus, hyperinsulinemia could directly promote erythrocytosis. Additionally, Hb regulates endothelial function by affecting the bioavailability of nitric oxide. The bioavailability of nitric oxide is impaired in many tissues, such as in the myocardium and coronary vasculature, with coexisting obesity and/or diabetes-related metabolic disorders [43,44,45,46]. Moreover, the Hb levels have been inversely associated with vascular endothelial function in type 2 diabetic patients [47]. Hb is also found to be inversely associated with adiponectin, a hormone that is released from adipose tissue that regulates lipid and glucose metabolism and is inversely associated with obesity and, especially, the amount of visceral fat [48]. Furthermore, higher Hb levels are associated with increased proinflammatory cytokines derived from adipose tissue in obese subjects with prediabetes [49]. Finally, it appears that visceral/abdominal obesity is the connecting factor between the Hb levels and insulin resistance [50]. 

A strong body of evidence suggests that increased sedentary time, which is often linked with obesity, is associated with an increased risk for increased overall mortality, type-2 diabetes, and cardiovascular disease, as well as cancer [17,51]. Less research has been done on the mechanistic details of sedentary time and disease risk focused on Hb and white blood cell counts. In the National Health and Nutrition Examination Survey (NHANES), higher levels of MVPA and less sedentary time were associated with lower white blood cell counts [17]. Similarly to the NHANES, in the ATTICA studies, physically active individuals with metabolic syndrome had lower inflammatory biomarkers such as WBC concentrations, compared to sedentary counterparts [24]. Additionally, replacing SB with MVPA seemed to improve the proinflammatory status, such as in WBC [25]. In our study, a higher sedentary time was associated positively with the WBC counts, which is in line with the previous studies. We also observed a favorable association between standing time and WBC, indicating lower WBC levels with longer standing time. However, an additional adjustment with the BMI in multivariable models diluted almost all significant correlations. Thus, this strongly suggest that body adiposity is a much stronger independent explanatory variable in predicting Hb and red blood cell characteristics, as well as the WBC count, than SB and PA characteristics. However, SB and PA are associated with overweightness and obesity, and they both have the potential to help in achieving and maintaining a normal body weight. Furthermore, even if Hb and other related variables are not affected, exercise and interruptions in SB have favorable effects on many traditional cardiovascular risk factors, as well as brain health [52,53,54].

In this study, breaks in sedentary time were positively correlated with Hb after adjusting with the BMI. This association is in contrast with a previous finding that uninterrupted sitting may acutely increase hemoglobin and hematocrit [26]. This is most likely because uninterrupted sitting may decrease the plasma volume, but individuals interrupting sitting with more frequent breaks may be fitter, and higher Hb is, in this case, connected with better aerobic fitness.

Physical inactivity and SB also contribute to renal dysfunction [20]. Plasma Cr is a breakdown product of muscle metabolism, which is cleared by the kidneys and commonly used as a marker of renal function. In our study, Cr was negatively correlated with BMI, WC and SBP and positively with breaks in the sedentary time. We can speculate that our participants might have had less muscle mass and relatively more adipose tissue than the average population, because they were physically inactive. At the same time, despite the overweight, the participants were relatively healthy, and secondary health outcomes such as renal dysfunction might not have taken place yet. 

Our study possesses many strengths, as well as some limitations that must be considered. The key strength of our study is the utilization of accelerometer-measured PA and sedentary time that were analyzed with validated methods. The benefit of measuring PA and SB by accelerometers is added accuracy and elimination of recall bias associated with self-reported data. In the current study, the participants were to use the accelerometers for four weeks consecutively, which is a longer period than many other similar studies have evaluated before. This might be beneficial, as it may represent the amount of PA that the participants actually do in their everyday life more truthfully. On the other hand, the average values of a longer period of time eradicates data from the individual variation in daily and overall intensity of PA and exercise. This variation of intensity may have effects on metabolism. The limitation of the current study is the observational setting, and therefore, randomized clinical trials aimed at lowering cardiometabolic risk are needed to evaluate the matter further. Another limitation is that we only used BMI and WC as indicators for body adiposity. Measurements of more detailed body composition indicators could have given us a more accurate understanding of the body adiposity. We did not measure adipose tissue-derived hormones or hormones affecting appetite and energy expenditure either [55], which could also affect adipose tissue and behaviors. Moreover, we did not measure other Hb-related factors, such as ferritin, erythropoietin or haptoglobin concentrations, which might have given further mechanistic insights into the matter. Further, we did not measure 2,3-diphosphoglycerate, which is an important molecule connected to Hb and affecting oxygen affinity beyond simply Hb concentrations, but it is unlikely that it has a major influence on the outcomes, as it is known to be little affected by acute or long-term exercise [56] and thus likely not affected by the SB or PA levels investigated in the present study either. Finally, higher Hb levels are usually favorable in terms of aerobic fitness [57,58], which was unfortunately not measured in the present study, and determining its role warrants further investigations in overweight and obese persons. Despite the Hb levels, matching of oxygen delivery precisely to the needs of the specific tissue (both spatially and temporally) is of utmost importance both in health and disease [59,60,61], and also, tissue distribution of oxygen warrants further studies in this population.

## 5. Conclusions

In the present study of working-aged, inactive, overweight and obese adults, we found that both the BMI and WC associated positively with Hb, WBC, RBC and HTC when adjusted for age and sex. Participants with lower Hb levels also had significantly lower SBP, HOMA-IR scores, LDL and triglyceride levels. All these findings suggest that Hb levels in the high end of normal range are associated with metabolic derangement and clustered around abdominal obesity. We also observed a positive association between SB and WBC in this population. Our findings reinforce the conception that SB increases the risk of chronic diseases, perhaps partly through unfavorable influence WBC, whereas lower sedentary time and higher PA would likely lead to healthier blood profiles and thereby reduce the risk of chronic diseases. However, when BMI was added to the statistical analysis, the association between SB and WBC turned nonsignificant. This suggests that obesity has an independent effect on WBC, unlike SB, and increasing the WBC levels might simply be a reflection of low-grade inflammation in the body. It is therefore likely that a lower level of obesity and less body fat are the main reasons for healthier blood profiles, and the reduction of body adiposity is an important way to mitigate the risks of chronic diseases. 

## Figures and Tables

**Table 1:** Characteristics of the study participants by sex. The results are reported as the mean (SD).

	Males	Females
n, (% of total)	42 (29)	102 (71)
Age, years	58.0 (6.0)	56.4 (6.7)
**Anthropometrics**		
BMI, kg/m^2^	31.8 (3.6)	31.7 (4.2)
Waist circumference, cm	116.3 (11.0)	106.7 (10.4) ***
**Blood parameters**		
WBC, 10^9^/L	6.6 (1.7)	6.1 (1.6)
RBC, 10^12^/L	5.2 (0.4)	4.7 (0.3) ***
HTC	45.9 (0)	42.2 (0) ***
Hb, g/L	155.9 (11.2)	140.3 (9.3) ***
MCV, fl	89.2 (4)	90.2 (3.7)
MCH, pg	30.4 (1.5)	30 (1.5)
TC, 10^9^/L	232.4 (43.1)	289.4 (78.6)
Cr, µmol/L	91.6 (12.6)	73.8 (11.1)
**Cardiometabolic risk factors**		
Systolic blood pressure, mmHg	149 (19)	147 (20)
Diastolic blood pressure, mmHg	91 (11)	90 (12)
Resting heart rate, bpm	70 (11)	71 (11)
BPL medication, n (%)	23 (55)	34 (33) *
CL medication, n (%)	8 (11)	11 (11)
f-Glucose, mmol/L	5.9 (0.7)	5.8 (0.9)
f-Insulin, µmol/L	16 (10)	12 (7) **
HOMA-IR	4.2 (3.0)	3.2 (2.4) *
HbA_1c_, mmol/mol	38 (5)	37 (6)
ALT, U/L	37 (20)	28 (14) **
AST, U/L	29 (10)	27 (7)
GGT, U/L	40 (19)	33 (33) **
Triglycerides, mmol/L	1.6 (0.9)	1.4 (0.8)
Cholesterol, mmol/L	5.0 (0.7)	5.4 (0.9) *
HDL-cholesterol, mmol/L	1.3 (0.3)	1.7 (0.4) ***
LDL-cholesterol, mmol/L	3.4 (0.7)	3.5 (0.9)
**Accelerometer measurements**		
Accelerometry, days	24 (5)	26 (4)
Wear time, h/day	14.3 (1.1)	14.4 (1.0)
Lying time, h/days	2.0 (1.1)	1.3 (0.7) ***
Sitting time, h/day	8.1 (1.4)	8.1 (1.1)
Sedentary time, h/day	10.1 (1.2)	9.4 (1.3) **
Sedentary proportion, %/day	71.0 (7.3)	65.4 (8.1) ***
Breaks in sedentary time, n/day	26 (7)	30 (8) **
Standing, h/day	1.4 (0.4)	2.2 (0.8) ***
Standing proportion, %/day	10.1 (2.9)	15.0 (5.0) ***
Daily steps	5408 (2288)	5206 (2046)
LPA, h/day	1.7 (0.6)	1.8 (0.5)
LPA proportion, %/day	11.7 (3.9)	12.8 (3.1)
MVPA, h/day	1.0 (0.4)	0.98 (0.4)
MVPA proportion, %/day	7.3 (2.9)	6.8 (2.5)
PA, h/day	2.7 (0.9)	2.8 (0.7)
PA proportion, %/day	19.0 (5.8)	19.6 (4.8)

Significant *p*-values; * *p* < 0.05, ** *p* < 0.01 and *** *p* < 0.001 for sex difference in the *t*-test (or Fisher’s exact test, when applicable). Abbreviations: BMI = body mass index, WBC = white blood cell count, RBC = red blood cell count, HTC = hematocrit, Hb = hemoglobin, MCV = mean cell volume, MCH = mean corpuscular hemoglobin, TC = thrombocytes, Cr = creatinine, BPL = blood pressure lowering, CL = cholesterol lowering, f = fasting, HOMA-IR = homeostatic model assessment for insulin resistance, HbA1c = hemoglobin A1c, ALT = alanine transaminase, AST = aspartate aminotransferase, GGT = gamma-glutamyl transferase; HDL = high-density lipoprotein, LDL = low-density lipoprotein, LPA = light physical activity; MVPA = moderate to vigorous physical activity and PA = physical activity (LPA and MVPA together).

**Table 2:** Heatmap of age- and sex-adjusted Pearson partial correlation coefficients between anthropometrics, cardiometabolic risk factors and common blood parameters (model 1).

	WBC _(E9/L)_	RBC _(E12/L)_	HTC	Hb _(g/L)_	MCV _(fl)_	MCH_(pg)_	TC _(E9/L)_	Cr _(µmol/L)_
**Anthropometrics**								
BMI	0.28 **	0.17 *	0.22 **	0.17 *	0.04	−0.02	0.08	−0.21 *
Waist (cm)	0.25 **	0.17 *	0.25 **	0.19 *	0.08	0.00	0.00	−0.21 *
**Cardiometabolic risk factors**								
Systolic blood pressure (mmHg)	0.02	0.22 **	0.20 *	0.17 *	−0.06	−0.05	0.06	−0.25 **
Diastolic blood pressure (mmHg)	0.04	0.23 *	0.19 *	0.14	−0.11	−0.12	0.10	−0.15
Resting heart rate (bpm)	0.02	0.04	0.11	0.02	0.08	−0.04	0.10	−0.09
Blood pressure medication	0.15	0.14	0.05	0.03	−0.16	−0.19	0.19 *	0.04
Cholesterol medication	0.03	0.10	0.11	0.07	−0.01	−0.02	0.01	−0.11
f-Glucose (mmol/L)	0.20 *	0.03	0.11	0.03	0.10	0.00	−0.05	−0.07
f-Insulin (mU/L)	0.29 **	0.16	0.16	0.16	−0.04	−0.03	0.05	−0.07
HOMA-IR	0.33 ***	0.17 *	0.20 *	0.17 *	0.00	−0.00	0.05	−0.07
HbA1c (mmol/mol)	0.10	0.06	0.09	−0.03	0.01	−0.13	−0.03	−0.07
ALT (U/L)	0.10	0.18 *	0.21 *	0.22 *	0.05	0.06	−0.07	−0.15
AST (U/L)	−0.08	0.09	0.07	0.09	−0.03	0.00	0.00	−0.08
GGT (U/L)	0.27 **	0.10	0.21 *	0.23 *	0.14	0.18 *	0.15	−0.20 *
Triglycerides (mmol/L)	0.37 ***	0.10	0.13	0.17 *	0.02	0.08	0.1	−0.02
Cholesterol (mmol/L)	0.09	0.06	0.14	0.14	0.09	0.10	0.05	0.01
HDL (mmol/L)	−0.18 *	−0.15	−0.17 *	−0.17	−0.00	−0.01	−0.02	−0.10
LDL (mmol/L)	0.06	0.09	0.20 *	0.19 *	0.14	0.12	−0.00	0.02

Significant *p*-values: * *p* < 0.05, ** *p* < 0.01 and *** *p* < 0.001. Colors indicate correlations (red-positive, blue-negative). Abbreviations: ALT = alanine transaminase, AST = aspartate aminotransferase, Cr = creatinine, f = fasting, GGT = gamma-glutamyl transferase, Hb = hemoglobin, HbA1c = hemoglobin A1c, HDL = high-density lipoprotein, HOMA-IR = homeostatic model assessment for insulin resistance, HTC = hematocrit, LDL = low-density lipoprotein, MCH = mean corpuscular hemoglobin, MCV = mean cell volume, RBC = red blood cell, TC = thrombocytes and WBC= white blood cells.

**Table 3:** Age-, sex- and BMI-adjusted linear mixed regression estimates (B values) between cardiometabolic risk factors and blood count parameters and Cr (model 2).

	WBC _(E9/L)_	RBC _(E12/L)_	HTC	Hb _(g/L)_	MCV _(fl)_	MCH _(pg)_	TC _(E9/L)_	Cr _(µmol/L)_
**Cardiometabolic risk factors**								
Systolic blood pressure, mmHg	2.75 × 10^−4^	3.05 × 10^−4^ *	2.19 × 10^−4^	7.50 × 10^−2^	−1.44 × 10^−2^	−3.25 × 10^−3^	0.15	−0.12 *
Diastolic blood pressure, mmHg	−4.63 × 10^−5^	5.77 × 10^−4^ *	3.91 × 10^−4^	0.11	−4.02 × 10^−2^	−1.56 × 10^−2^	0.53	−0.10
Resting heart rate, bpm	6.52 × 10^−5^	8.91 × 10^−5^	2.49 × 10^−4^	2.23 × 10^−2^	2.86 × 10^−2^	−3.62 × 10^−3^	0.52	−5.88 × 10^−2^
Blood pressure medication	−1.37 × 10^−2^	−3.75 × 10^−3^	−7.02 × 10^−4^	−0.10	0.67 *	0.29 *	−13.38 *	−0.75
Cholesterol medication	−6.02 × 10^−4^	−3.42 × 10^−3^	−3.34 × 10^−3^	−0.60	7.60 × 10^−2^	3.19 × 10^−2^	−1.13	1.82
f-Glucose, mmol/L	1.26 × 10^−2^	−6.26 × 10^−4^	1.46 × 10^−3^	−0.36	0.37	−9.02 × 10^−3^	−7.63	0.13
f-Insulin, mU/L	2.60 × 10^−3^	3.93 × 10^−4^	1.68 × 10^−4^	0.12	−4.18 × 10^−2^	3.50 × 10^−3^	8.24 × 10^−2^	8.79 × 10^−2^
HOMA-IR	8.26 × 10^−3^ *	1.05 × 10^−3^	4.89 × 10^−4^	0.28	−0.10	1.81 × 10^−2^	−0.75	0.31
HbA1c, mmol/mol	3.23 × 10^−4^	1.61 × 10^−4^	1.88 × 10^−4^	−0.11	3.35 × 10^−4^	−2.88 × 10^−2^	−0.49	−7.85 × 10^−2^
ALT, U/L	2.99 × 10^−5^	1.39 × 10^−4^	1.10 × 10^−4^	5.48 × 10^−2^	3.29 × 10^−3^	3.45 × 10^−3^	−0.39	−7.56 × 10^−2^
AST, U/L	−1.88 × 10^−3^	1.76 × 10^−4^	1.33 × 10^−5^	2.64 × 10^−2^	−3.06 × 10^−2^	5.12 × 10^−3^	−5.60 × 10^−3^	−8.59 × 10^−2^
GGT, U/L	4.56 × 10^−4^	7.38 × 10^−5^	9.82 × 10^−5^	4.69 × 10^−2^	4.92 × 10^−3^	5.53 × 10^−3^	0.28	−5.75 × 10^−2^
Triglycerides, mmol/L	4.15 × 10^−2^ ***	1.57 × 10^−3^	1.42 × 10^−3^	1.38	−0.14	0.12	9.98	1.19
Cholesterol, mmol/L	1.43 × 10^−2^	2.76 × 10^−3^	4.90 × 10^−3^	1.81	0.44	0.17	4.63	−6.47 × 10^−2^
HDL, mmol/L	−2.66 × 10^−2^	−9.52 × 10^−3^	−9.54 × 10^−3^	−3.59	4.91 × 10^−2^	−0.12	5.23	−4.49
LDL, mmol/L	1.17 × 10^−2^	2.98 × 10^−3^	5.92 × 10^−3^ *	2.07 *	0.61	0.22	−1.09	0.31

Significant *p*-values: * *p* < 0.05 and *** *p* < 0.001. Abbreviations: ALT = alanine transaminase, AST = aspartate aminotransferase, Cr = creatinine, f = fasting, GGT = gamma-glutamyl transferase, Hb = hemoglobin, HbA1c = hemoglobin A1c, HDL = high-density lipoprotein, HOMA-IR = homeostatic model assessment for insulin resistance, HTC = hematocrit, LDL = low-density lipoprotein, MCH = mean corpuscular hemoglobin, MCV = mean cell volume, RBC = red blood cell, TC = thrombocytes and WBC= white blood cells.

**Table 4:** Heatmap of age- and sex-adjusted Pearson partial correlation coefficients between sedentary behavior, physical activity and blood count parameters and Cr (model 1).

	WBC _(E9/L)_	RBC _(E12/L)_	HTC	Hb _(g/L)_	MCV _(fl)_	MCH_(pg)_	TC _(E9/L)_	Cr _(µmol/L)_
**Sedentary behavior**								
Lying time (h/day)	0.18 *	0.26 **	0.20 *	0.19 *	−0.18 *	−0.13	0.08	−0.13
Sitting time (h/day)	−0.05	−0.06	−0.03	−0.05	0.07	−0.00	−0.09	0.05
Sedentary time (h/day)	0.07	0.07	0.07	0.05	−0.03	−0.07	−0.00	−0.06
Sedentary proportion (%/day)	0.17 *	0.03	0.02	0.01	−0.04	−0.07	0.02	−0.09
**Physical activity**								
Breaks in sedentary time (n/day)	−0.08	0.10	0.08	0.15	−0.02	−0.08	0.01	0.17 *
Standing (h/day)	−0.19 *	−0.01	−0.07	−0.08	−0.05	−0.05	−0.05	0.17
Standing proportion (%/day)	−0.14	−0.02	−0.09	−0.09	−0.06	−0.04	−0.05	0.16
Steps (number/day)	−0.11	−0.06	−0.07	−0.04	−0.00	0.05	0.09	0.02
LPA (h/day)	−0.15	0.05	0.16	0.14	0.17	0.15	−0.03	0.03
LPA proportion (%/day)	−0.13	0.02	0.14	0.13	0.18 *	0.17	−0.04	0.02
MVPA (h/day)	−0.10	−0.03	−0.03	−0.02	−0.00	0.04	0.07	0.04
MVPA proportion (%/day)	−0.09	−0.05	−0.05	−0.04	0.00	0.05	0.07	0.02
PA, h/day	0.02	−0.04	−0.02	0.00	0.09	0.08	0.09	−0.1
PA, %/day	0.03	−0.06	−0.02	0.01	0.12	0.12	0.08	−0.09
MET peak	−0.10	−0.16	−0.16	−0.13	0.03	0.06	−0.03	0.09
MET mean	−0.13	−0.07	−0.03	−0.00	0.07	0.12	0.03	0.02

Significant *p*-values: * *p* < 0.05, ** *p* < 0.01. Colors indicate correlations (red-positive, blue-negative). Abbreviations: Cr = creatinine, Hb = hemoglobin, HTC = hematocrit, LPA = light physical activity; MCH = mean corpuscular hemoglobin MCV = mean cell volume, MET = metabolic equivalent, MPA = moderate physical activity; MVPA = moderate to vigorous physical activity, PA = physical activity (LPA and MVPA together), RBC = red blood cell, TC = thrombocytes and WBC = white blood cell.

**Table 5:** Age-, sex- and BMI-adjusted linear mixed regression estimates (B values) between sedentary behavior, physical activity and common blood count parameters and Cr (model 2).

	WBC _(E9/L)_	RBC _(E12/L)_	HTC	Hb _(g/L)_	MCV _(fl)_	MCH _(pg)_	TC _(E9/L)_	Cr _(µmol/L)_
**Sedentary behavior**								
Lying time, h/day	2.00 × 10^−2^	7.77 × 10^−3^ *	4.77 × 10^−3^	1.90	−0.74	−0.17	8.99	−1.58
Sitting time, h/day	−5.98 × 10^−3^	−2.24 × 10^−3^	−1.27 × 10^−3^	−0.64	0.21	−1.26 × 10^−2^	−4.95	0.38
Sedentary time, h/day	2.88 × 10^−3^	1.18 × 10^−3^	8.18 × 10^−4^	0.21	−0.11	−6.88 × 10^−3^	5.48 × 10^−5^	−0.31
Sedentary proportion, %/day	0.16	5.60 × 10^−4^	−6.78 × 10^−3^	−2.05	−2.51	−0.11	2.10 × 10^−2^	−5.53
**Physical activity**								
Breaks in sedentary time, n/day	1.15 × 10^−4^	6.08 × 10^−4^	5.20 × 10^−4^	0.26 *	−2.48 × 10^−3^	1.24 × 10^−3^	8.73 × 10^−4^	0.15
Standing, h/day	−1.65 × 10^−2^	1.33 × 10^−3^	−1.04 × 10^−3^	−0.64	−0.32	−1.13 × 10^−2^	−1.51 × 10^−3^	1.84
Standing proportion, %/day	−0.22	3.49 × 10^−3^	−2.99 × 10^−2^	−14.08	−4.52	−0.14	−7.57 × 10^−2^	29.73
Steps, number/day	−1.66 × 10^−6^	−1.28 × 10^−7^	−3.01 × 10^−8^	5.68 × 10^−5^	2.82 × 10^−5^	3.40 × 10^−6^	4.96 × 10^−6^	−2.68 × 10^−4^
LPA, h/day	−2.46 × 10^−2^	3.05 × 10^−3^	8.43 × 10^−3^	3.04	1.26 *	4.34 × 10^−2^	−1.88 × 10^−3^	−0.82
LPA proportion, %/day	−0.37	1.66 × 10^−2^	0.10	38.23	20.14 *	0.68 *	−0.11	−13.44
MVPA, h/day	−1.10 × 10^−2^	−7.14 × 10^−4^	−9.10 × 10^−4^	−1.70 × 10^−2^	−2.24 × 10^−2^	1.36 × 10^−2^	2.18 × 10^−2^	−0.92
MVPA proportion, %/day	−0.14	−3.69 × 10^−2^	−3.45 × 10^−2^	−7.18	0.63	0.23	0.24	−16.86
PA, h/day	3.88 × 10^−3^	−1.30 × 10^−3^	3.59 × 10^−3^	1.37	0.56	2.27 × 10^−2^	4.13 × 10^−3^	−1.00
PA, %/day	6.85 × 10^−2^	−2.86 × 10^−2^	3.86 × 10^−2^	15.54	9.25	0.37	1.03 × 10^−2^	−14.79
MET peak	−5.17 × 10^−3^	−8.11 × 10^−3^	−5.44 × 10^−3^	−1.58	0.50	2.02 × 10^−2^	−2.13 × 10^−3^	0.96
MET mean	−6.97 × 10^−2^	−1.21 × 10^−2^	2.38 × 10^−3^	2.41	3.32	0.16	2.25 × 10^−2^	−5.24

Significant *p*-values: * *p* < 0.05. Abbreviations: Cr = creatinine, Hb = hemoglobin, HTC = hematocrit, LPA = light physical activity; MCH = mean corpuscular hemoglobin MCV = mean cell volume, MET = metabolic equivalent, MPA = moderate physical activity; MVPA = moderate to vigorous physical activity, PA = physical activity (LPA and MVPA together), RBC = red blood cell, TC = thrombocytes and WBC = white blood cell.

## Data Availability

Data may be obtained upon reasonable request.

## Abbreviations

ALT	Alanine aminotransferase
APE	angle of posture estimation
BMI	body mass index
Cr	creatinine
DBP	diastolic blood pressure
Hb	hemoglobin
HOMA-IR	homeostatic model assessment for insulin resistance
HTC	hematocrit
LPA	light physical activity
MAD	mean amplitude deviation
MCH	mean corpuscular hemoglobin
MCV	mean cell volume
MET	metabolic equivalent
MVPA	moderate to vigorous physical activity
PA	physical activity
RBC	red blood cell
SB	sedentary behavior
SBP	systolic blood pressure
TC	thrombocyte
WBC	white blood cell
WC	waist circumference
